# Safety and efficacy of catheter ablation of para‐Hisian accessory pathway via a direct superior vena cava approach: A multicenter study

**DOI:** 10.1002/clc.24180

**Published:** 2023-10-27

**Authors:** Chanjuan Chai, Qi Sun, Xiao‐Gang Guo, Jian‐Du Yang, Hai‐Yang Xie, Jian Ma, Hui‐Qiang Wei, Jie Yu

**Affiliations:** ^1^ Department of Cardiology The Second Hospital of Shanxi Medical University Taiyuan China; ^2^ State Key Laboratory of Cardiovascular Disease Arrhythmia Center, Fuwai Hospital, National Center for Cardiovascular Diseases, Chinese Academy of Medical Sciences and Peking Union Medical College Beijing China; ^3^ Departent of Cardiology Sun Yat‐Sen Memorial Hospital Sun Yat‐Sen University Guangzhou China; ^4^ Department of Cardiology Guangdong Cardiovascular Institute, Guangdong Provincial People's Hospital (Guangdong Academy of Medical Sciences) Southern Medical University Guangzhou China; ^5^ Department of Cardiology Yantaishan Hospital Yantai China

**Keywords:** accessory pathway, catheter ablation, para‐Hisian, radiofrequency, superior vena cava

## Abstract

**Background:**

Radiofrequency (RF) catheter ablation of para‐Hisian accessory pathways (APs) can be challenging due to proximity to the conduction system.

**Methods:**

A total of 30 consecutive patients with para‐Hisian AP were enrolled for ablation in three centers, 12 (40%) of whom had previously failed attempted ablation from the inferior vena cava (IVC) approach. Ablation was preferentially performed using a superior approach from the superior vena cava (SVC) in all patients.

**Results:**

The para‐Hisian AP was eliminated from the SVC approach in 28 of 30 (93.3%) patients. In the remaining two patients, additional ablation from IVC was required to successfully eliminate the AP. There were two patients experienced reversible complete atrial‐ventricular block and PR prolongation during the first RF application. Long‐term freedom from recurrent arrhythmia was achieved in 29 (96.7%) patients over a mean follow‐up duration of 15.6 ± 4.6 months.

**Conclusion:**

Catheter ablation of para‐Hisian AP from above using a direct SVC approach is both safe and effective, and should be considered especially in patients who have failed conventional ablation attempts from IVC approach.

## Graphical abstract

Ablation was preferentially performed using a superior vena cava (SVC) approach in 30 patients with para‐Hisian accessory pathways (APs) in three centers. Catheter ablation of para‐Hisian AP from above using a direct SVC approach is both safe and effective, with a favorable long‐term outcome.

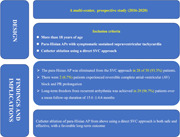

## 1 INTRODUCTION

Radiofrequency (RF) catheter ablation has been established as an effective therapy of accessory pathways (APs).^1^, ^2^, ^3^ However, para‐Hisian APs remain challenging to eliminate with catheter ablation due to the proximity of the APs to the normal conduction system.^4^ Recurrence rates and the risk of complete atrioventricular (AV) block are relatively higher in patients undergoing ablation for para‐Hisian APs. The conventional approach to the ablation of para‐Hisian APs involves an inferior approach with femoral venous access with the advancement of the ablation catheter from below through the inferior vena cava (IVC). In some patients, however, safe elimination of these APs can be difficult to achieve from the standard IVC approach, and alternative approaches from the superior vena cava (SVC) approach or noncoronary cusp (NCC) can be successful.^5^, ^6^, ^7^ A superior approach with internal jugular, axillary, or subclavian venous access and advancement of the ablation catheter through the SVC to the right atrial (RA) has been utilized as an alternative strategy to target para‐Hisian APs.^7^ However, there were single‐center studies with small sample sizes. The safety and efficacy of catheter ablation of para‐Hisian AP via a direct SVC approach remains unknown. We aimed to examine the outcomes and complications of ablating para‐Hisian APs using a direct SVC approach.

## 2 METHODS

### 2.1 Ethical statement

Ethical approvals were obtained from the Fuwai Hospital, Yantaishan Hospital, and Second Hospital of Shanxi Medical University Research Ethics Committees. The Institutional Review Board approved the study protocol and all patients signed written informed consent before the intervention, including the full set of risk‐informed consent and information use consent for scientific purposes.

### 2.2 Study population

We screened our institutional ablation database to identify all patients who underwent RF catheter ablation of APs between January 2016 and July 2020 at three centers. The inclusion criteria were as follows: patients more than 18 years of age, who had para‐Hisian APs, presented with symptomatic sustained supraventricular tachycardia and underwent RF catheter ablation using the SVC approach. The study was approved by the institutional review board and written informed consent was obtained from each patient before the procedure.

### 2.3 Electrophysiological study

All antiarrhythmic drugs were discontinued for at least five half‐lives before the study. All procedures were performed under conscious sedation with local anesthesia. Two quadripolar catheters were advanced to the His bundle (HB) region and right ventricular apex via the femoral veins. A decapolar catheter was positioned in the coronary sinus (CS) via the right internal jugular vein. The proximal bipole of this catheter was placed at the ostium of the CS. Data were recorded and stored simultaneously by a digital multichannel system (LabSystem PRO; Bard Electrophysiology). Bipolar intracardiac electrograms were filtered between 30 and 500 Hz, and unipolar intracardiac electrograms were filtered at 0.5–500 Hz. Standard atrial and ventricular pacing protocols were performed with rapid atrial pacing, up to double atrial extrastimulus testing, ventricular overdrive pacing, and single ventricular extrastimulus testing. Following documenting narrow QRS tachycardias, differential maneuvers were performed. Localization and identification of the APs was achieved with meticulous mapping of the atrial and/or ventricular activation pattern.

The APs were considered to be para‐Hisian when a discernible HB potential was recorded at the site of earliest atrial activation during the retrograde AP conduction or following ablation of a manifest AP with the disappearance of ventricular pre‐excitation.

### 2.4 Mapping and ablation

Activation mapping was initially performed either during tachycardia, during retrograde AP conduction with right ventricular pacing, or during sinus rhythm (in the presence of manifest pre‐excitation) using a 4‐mm tip ablation catheter placed via a femoral vein. After confirmation of the diagnosis of the tachycardia and the presence of a para‐Hisian AP, the ablation catheter was removed from the body and inserted through the right internal jugular vein through the SVC into the right atrium. In all cases, ablation energy was initiated at 15 W and titrated up to 30–35 W targeting goal impedance drop of 8–10 Ω. During RF delivery, the PR interval, the presence of junctional beats, and catheter movement were continuously monitored. If after AP conduction persisted after 10 seconds of RF delivery at a specific location, RF delivery was ceased and further mapping was performed. If RF failed to terminate tachycardia or eliminate AP conduction via the SVC approach, ablation was then attempted using the conventional strategy from an IVC approach. Acute success was defined as the elimination of AP ventriculoatrial conduction or loss of antegrade AP conduction with the elimination of ventricular pre‐excitation.

### 2.5 Postprocedural management and follow‐up

After the procedure, 12‐lead electrocardiogram (ECG) was recorded immediately in all patients. Patients were monitored overnight on telemetry and discharged the subsequent day with aspirin for 1 month. Routine follow‐up included surface ECG and Holter monitoring for all patients with symptoms suggestive of recurrent arrhythmia.

### 2.6 Statistical analysis

All continuous variables are presented as mean ± SD and categorical variables are expressed as numbers and percentages. Categorical variables were compared using *χ* ^2^ analysis. Continuous variables were compared using the Student *t*‐test or Mann–Whitney *U* test, depending on data distribution. A two‐tailed value of *p* < .05 was considered statistically significant. All statistical analyses were performed using SPSS 19.0 (SPSS Inc.).

## 3 RESULTS

### 3.1 Patient characteristics

Among 455 consecutive patients who underwent RF catheter ablation of APs between January 2016 and July 2020 in three centers, 30 consecutive patients with para‐Hisian AP who underwent attempted ablation using a direct SVC approach were included. The previous ablation had been performed in 12 (40%) patients. All patients were symptomatic with paroxysmal palpitations. Three‐dimensional electroanatomic mapping was utilized in all patients. Of the 30 study patients, 12 (40%) had evidence of manifest ventricular pre‐excitation (10 had persistent pre‐excitation, two had intermittent pre‐excitation). One patient had multiple APs: a manifest RA appendage AP in addition to the manifest para‐Hisian AP. One patient had hypertrophic cardiomyopathy. One patient had recurrent syncope caused by fast ventricular rate via antegrade AP conduction during the paroxysmal atrial fibrillation. The mean cycle length of the tachycardia mediated by the para‐Hisian AP was 358.4 ± 61.1 ms. Baseline characteristics of the patients are presented in Table 1.

**Table 1:** Baseline characteristics.

Patients	*N* = 30
Age (years)	27.2 ± 4.6
Male (%)	20 (66.7%)
Failed previous ablation attempt, *n* (%)	12 (40%)
Manifest ventricular pre‐excitation, *n* (%)	12 (40%)
Structure heart disease, *n* (%)	1 (3.3%)
Mean cycle length of the tachycardia (ms)	358.4 ± 61.1

### 3.2 Catheter ablation

**Figure 1:**
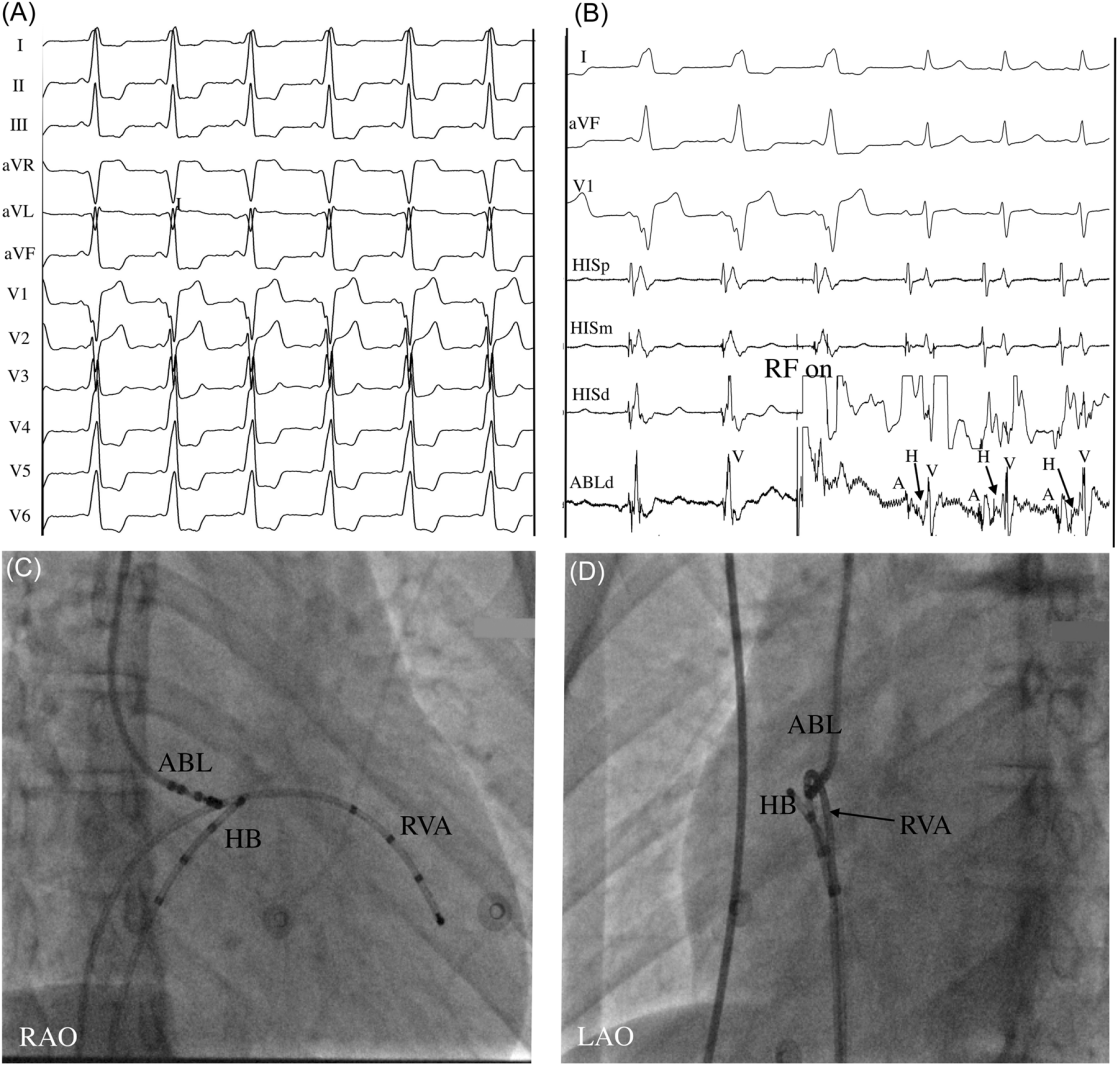
Patient 5 with a manifest para‐Hisian AP. (A) Twelve‐lead ECG shows positive delta wave in leads I, II, aVL, aVF and precordial QRS transition at lead V_3_. (B) The tip of the ablation catheter is positioned at the para‐Hisian region, and a low‐amplitude atrial potential and early large ventricular potential is recorded (first three beats). After RF energy delivery, loss of AP conduction was observed with a recorded His bundle potential on the ablation catheter (last three beats). (C, D) RAO and LAO fluoroscopic images of ablation catheter position. ABL, ablation catheter; AP, accessory pathway; d, distal; ECG, electrocardiogram; HB, His bundle; LAO, left anterior oblique; m, middle; p, proximal; RAO, right anterior oblique; RF, radiofrequency; RVA, right ventricular apex.

Ablation from the SVC approach successfully eliminated the para‐Hisian APs in 28 (93.3%) of 30 patients (Figure 1). A mechanical bump of AP occurred in one patient during manipulation of the ablation catheter, resulting in temporary loss of AP conduction. In two patients, ablation using a SVC approach failed and an inferior approach from the IVC was required to successfully eliminate the para‐Hisian AP. The mean amplitude of local atrial and ventricular electrograms at the successful targets were 0.26 ± 0.10 and 1.31 ± 0.80 mV, respectively. The mean number of RF applications was 2.5 ± 0.9, and the mean time from the onset of the successful RF lesion to the elimination of AP conduction was 3.2 ± 3.9 seconds. The total RF time was 135 ± 17 seconds. The mean procedural duration and fluoroscopic time were 94 ± 25 and 9.3 ± 1.6 minutes, respectively (Table 2).

**Table 2:** Electrophysiological characteristics and ablation results.

Electrophysiological characteristics	*N* = 30
Time to loss of AP (s)	3.2 ± 3.9
Mean amplitude of local atrial electrograms at target site (mV)	0.26 ± 0.10
Mean amplitude of local ventricular electrograms at target site (mV)	1.31 ± 0.80
Mean number of RF applications, *n*	2.5 ± 0.9
Ablation results	
Successful ablation from SVC approach, *n* (%)	28 (93.3%)
Junctional rhythm during RF ablation, *n* (%)	4 (13.3%)
AV block, *n* (%)	2 (6.7%)
Total RF time (s)	135 ± 17
Procedural time (min)	94 ± 25
Fluoroscopic time (min)	9.3 ± 1.6

Abbreviations: AP, accessory pathway; AV, atrioventricular; RF, radiofrequency; SVC, superior vena cava.

### 3.3 Complications

**Figure 2:**
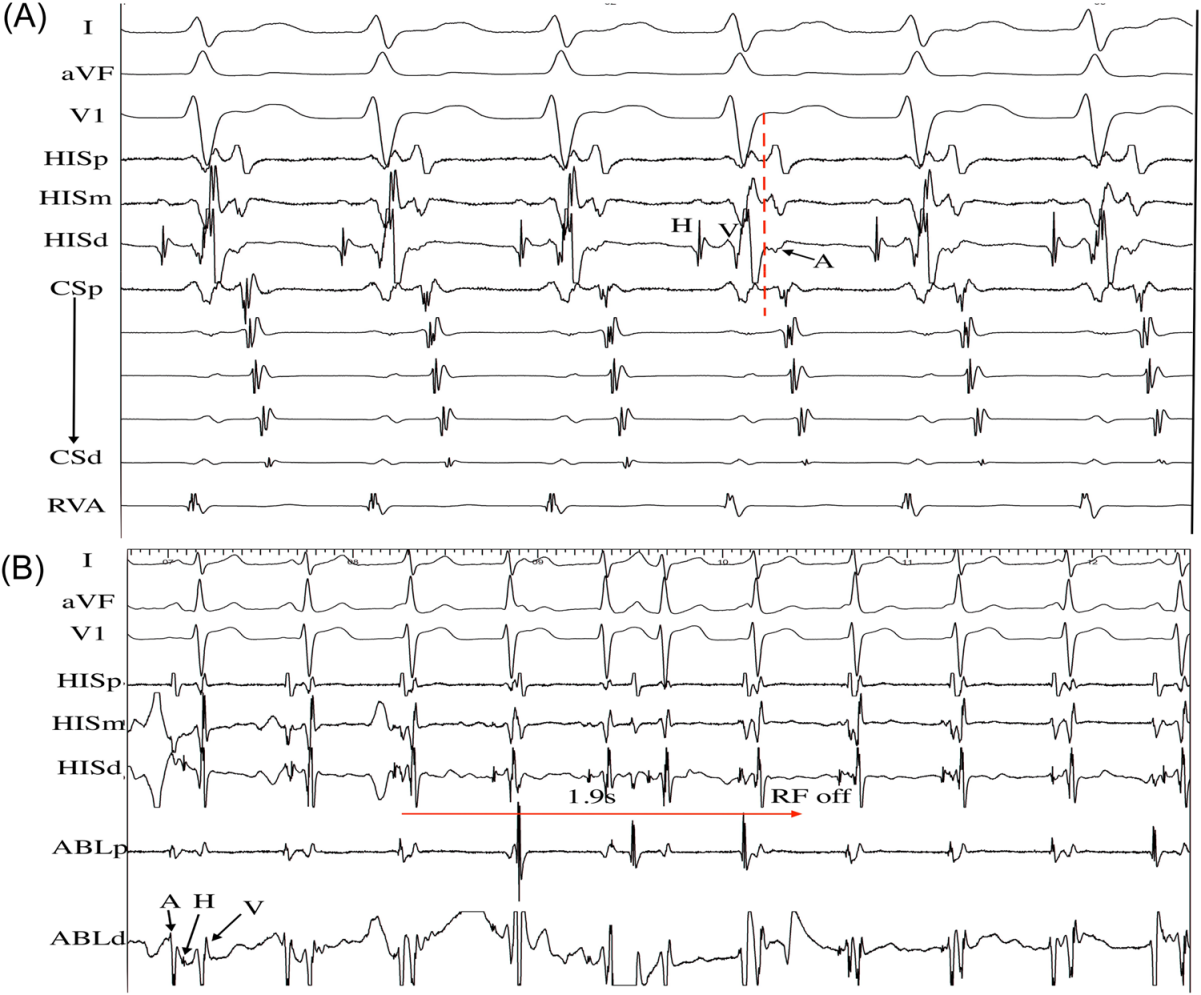
Patient 2 with a concealed para‐Hisian AP. (A) Intracardiac electrograms during the atrioventricular reentrant tachycardia showed that the earliest atrial potential was located in the distal HIS catheter. (B) The ablation catheter was positioned at the para‐Hisian region, and a large atrial and ventricular potential was recorded (first two beats). Junctional rhythm was observed at 6 seconds of ablation, and RF power delivery was interrupted after 1.9 seconds. Sinus rhythm recovered after three junction beats. ABL, ablation catheter; AP, accessory pathway; CS, coronary sinus; d, distal; HB, His bundle; m, middle; p, proximal; RF, radiofrequency; RVA, right ventricular apex.

Junctional rhythm during RF ablation was observed in four patients. In all four patients, ablation was discontinued immediately with junctional rhythm and AV conduction remained intact (Figure 2). Antegrade AP conduction was only transiently eliminated in two patients after four ablation attempts, after which the SVC approach was abandoned and ablation was performed via an IVC approach resulting in successful elimination of the APs without complication. There were two patients experienced reversible complete AV block and PR prolongation during the first RF application. None of the patients developed permanent complete AV block or impairment of AV conduction requiring a permanent pacemaker implant.

### 3.4 Follow‐up

After a mean follow‐up period of 15.6 ± 4.6 months after ablation, 29 (96.7%) patients had no recurrent tachycardia. The one patient with recurrent tachycardia underwent repeat ablation and successful ablation was achieved.

## 4 DISCUSSION

The results of our multi‐center study suggest that ablation of para‐Hisian APs via a direct SVC approach is both safe and effective, with a favorable long‐term outcome. Ablation from the SVC approach should be considered especially for those refractory to conventional attempts.

Ablation of para‐Hisian APs can be extremely challenging due to proximity to the normal conduction system and the risk of causing iatrogenic AV block. In the majority of studies, ablation of para‐Hisian APs has typically been performed via an IVC approach,^7^ with a high acute success rate. However, rates of tachycardia recurrence and procedural complications after RF ablation of para‐Hisian AP remain relatively high compared versus APs from other regions.^8^, ^9^ In the present study, we demonstrated a low rate (3.3%) of long‐term recurrence and no permanent iatrogenic damage to the AV node, supporting the notion that an SVC approach can be a both effective and safe strategy to treat para‐Hisian APs.

Para‐Hisian APs can also be successfully targeted with ablation from the NCC.^5^, ^6^ Xu et al.^7^ have previously described their experience with ablation of para‐Hisian APs from the NCC in 12 patients and reported that ablation from the NCC as a vantage point was successful in eliminating the para‐Hisian AP in 11 (91.7%) of 12 cases. Importantly, ablation from the NCC via a retrograde aortic approach requires femoral arterial access and contrast angiography of the aortic root needs to be performed during the procedure.^10^ The power required to achieve success was higher (30–40 W) and the duration of RF to eliminate AP conduction block was longer (>20–30 seconds in most patients). Furthermore, although uncommon, iatrogenic complete heart block has been described as a complication of ablation from the NCC.^11^ Cryoablation is another strategy which has the potential to minimize the risk of AV nodal injury and to improve catheter stability during ablation. However, acute and long‐term success rates with cryoablatoin tend to be lower than RF ablation.^12^, ^13^ Tuzcu et al.^14^ reported that the immediate success rate in children using the cryoablation was only 73%, with a recurrent rate of 24%.

Ablation of para‐Hisian APs using a superior approach has been previously described in several studies.^2^, ^9^, ^14^ In 1991, Jackman et al.^2^ first described 13 cases of anteroseptal APs ablated from subclavian approach. Subsequently, Mandapati et al.^5^ and DiLorenzo et al.^15^ reported high rates of success with ablation of anteroseptal APs from the right internal jugular vein and suggested that the SVC approach might be the preferred approach in pediatric patients. However, there were single‐center studies with small sample sizes. In current study, AP conduction was successfully ablated via the SVC approach in 28 of 30 (93.3%) patients without compromising AV conduction, which is consistent with the above‐mentioned studies. During the electrophysiological study, the ventricular and atrial electrograms were always fused during retrograde or antegrade AP conduction. Additionally, the amplitude of the atrial potential at the target region tended to be much smaller than the ventricular potential, indicating a safer ablation target which was closer to the ventricular side. The average time from the ablation start to the AP conduction block was 3.2 ± 3.9 seconds. Therefore, RF ablation via the SVC approach is a reasonable primary strategy of a secondary option in cases where the conventional attempts fail. In our experience, catheter stability was improved with an approach from internal jugular vein or subclavian vein even without a long sheath because this approach allowed the tip to be hooked with stable tissue contact along the ventricular side of the tricuspid valve. Additionally, the catheter manipulation and achieving adequate contact when using an SVC approach is no more difficult than with the traditional IVC approach. Some technical tips to achieve success include gentle torque on the mapping catheter to approach the ventricular side of para‐Hisian region and to aim to achieve a small atrial and large ventricular electrogram at the target site.

## 5 LIMITATIONS

There are several limitations in our study. Further randomized studies with larger sample sizes may be needed to confirm our findings. In our study, we did not attempt ablation from the NCC or use cryoablation before attempting the SVC approach. Furthermore, the SVC approach is a need to place an internal jugular vein or subclavian access. Not all centers place a catheter from the SVC and this would add an additional step and separate access location.

## 6 CONCLUSION

Ablation of para‐Hisian APs using a direct SVC approach can be effective both at the initial procedure and during repeat procedures after failed attempted ablation via a conventional IVC approach. This approach is both safe and effective with a favorable long‐term outcome.

## CONFLICT OF INTEREST STATEMENT

The authors declare no conflict of interest.

## Data Availability

The data used to support the findings of this study are available from the corresponding author upon request.
